# Leuprolide Acetate Promotes Sensory Recovery and Modulates Dorsal Root Ganglion Responses After Sciatic Nerve Transection in Rats

**DOI:** 10.3390/brainsci16030332

**Published:** 2026-03-20

**Authors:** Irma Hernández-Jasso, Denisse Calderón-Vallejo, José Ávila-Mendoza, David Epardo, Jerusa E. Balderas-Márquez, Carlos Arámburo, J. Luis Quintanar, Carlos G. Martínez-Moreno

**Affiliations:** 1 Departamento de Fisiología y Farmacología, Centro de Ciencias Básicas, Universidad Autónoma de Aguascalientes, Aguascalientes 20100, Mexico; irma.hernandez@edu.uaa.mx (I.H.-J.); denisse.calderon@edu.uaa.mx (D.C.-V.); 2 Departamento de Neurobiología Celular y Molecular, Instituto de Neurobiología, Campus Juriquilla, Universidad Nacional Autónoma de México, Querétaro 76230, Mexico; javila@comunidad.unam.mx (J.Á.-M.); epardodavid@comunidad.unam.mx (D.E.); jereli.balderasm@comunidad.unam.mx (J.E.B.-M.); aramburo@unam.mx (C.A.)

**Keywords:** leuprolide acetate, sciatic nerve, DRG, neuroregeneration, peripheral nerve injury, sensory function

## Abstract

**Highlights:**

**What are the main findings?**

• LA promotes selective recovery of cold sensitivity after sciatic nerve transection.
• LA enhances retrograde axonal transport after sciatic nerve transection.
• LA attenuates reactive gliosis and macrophage activation in dorsal root ganglia after sciatic nerve transection.
• LA modulates the expression of key pro-regenerative genes in peripheral nerve injury.

**What is the implication of the main finding?**

• The results suggest that LA could be a promising therapeutic strategy to improve sensory recovery and neuroregeneration following peripheral nerve injury.

**Abstract:**

**Background/Objectives**: Sciatic nerve injuries are among the most common classes of peripheral nerve harm and have a strong impact on quality of life, as well as a significant negative economic impact for patients, society, and governments, since they represent a frequent cause of work-related disabilities and sick leave applications. Following nerve injury, neurons, Schwann, and satellite cells undergo marked changes in phenotype, metabolic activity, neuronal survival, nervous transmission, and an exacerbated activation of the inflammatory response. Leuprolide acetate (LA), a clinically available agonist of gonadotropin-releasing hormone (GnRH), has shown clear neurotrophic properties and is considered a novel potential candidate for treating neural injuries, including sciatic nerve pathologies. This study aimed to analyze the effect of LA treatment on sensory function and dorsal root ganglia (DRG) changes in a rat sciatic nerve full-transection (SNT) model. **Methods**: Variations in cold and heat sensitivity were assessed using the thermal plate test, while DRG tissue sections were examined for modifications in reactive gliosis by immunofluorescence analysis, and axonal transport using a retrograde tracer. Also, changes in the expression of pro-regenerative genes *Stat3*, *Socs3*, *Fos*, *Jun*, *Atf4*, and *Limk1* were quantified by qPCR. **Results**: Our results showed that LA treatment exerted a distinct neurotrophic effect, since it promoted the specific recovery of cold sensitivity, improved axonal transport, regulated the inflammatory response, and modulated the exacerbated expression of pro-regenerative genes in the SNT model. **Conclusions**: These findings indicate that LA therapy may have the potential to improve sensory recovery in patients with sciatic nerve injuries.

## 1. Introduction

Sciatic nerve (SN) pathologies include ischemia/hypoxia, trauma, mechanical compression, and bone fractures as primary etiological factors leading to functional and structural nerve damage [1,2,3]. It is important to note that the sciatic nerve originates in the spinal cord, and the sensory pathway includes the dorsal root ganglia (DRG) as a relay before re-entering the central nervous system [4]. DRG are simple neural structures containing the cell bodies of sensory neurons; their axons, together with motor neuron axons, form the sciatic nerve [5,6,7].

Following SN injury, important modifications are induced in cell metabolism, neuronal survival processes, electrochemical impulse transmission, and the activation of the inflammatory response, which is mediated mainly by glial cells and peripheral immune cells that infiltrate the nerves and the central nervous system (CNS) [8,9]. Initially, during the acute and subacute phases of the lesion, the interaction between satellite cells, infiltrating immune cells, and sensory neurons is able to activate mechanisms that promote axonal neuroprotection and regeneration, which could eventually lead to functional reinnervation; this is a specific characteristic of peripheral nerves [10,11,12]. However, existing therapeutic options often fail to achieve complete restoration due to inflammation, glial scarring, and misdirection of regenerating sensory axons, resulting in neuropathic pain and compromised sensory and motor capabilities [13,14]. Therefore, innovative experimental approaches are being explored to develop effective strategies for functional nerve regeneration after damage.

Neurotrophic factors are intimately involved in neuroregeneration in the CNS, peripheral nerves, and innervated tissues, including classical neurotrophins such as brain-derived neurotrophic factor (BDNF), ciliary neurotrophic factor (CNTF), and nerve growth factor (NGF) [15,16]. In the peripheral nervous system, neurotrophic factors play critical roles in the survival, differentiation, tissue homeostasis, and regeneration of DRG neurons, Schwann cells, and even stromal cells [17,18,19]. It is important to note that Schwann cells are key players during nerve regeneration, as they guide axonal regrowth [20].

Gonadotropin-releasing hormone (GnRH) is a decapeptide that was initially described in the hypothalamus, although now it is well established that it is produced in many tissues and cell types [21,22]. GnRH exerts its effects by binding to its receptor (GnRH-R); in the pituitary, this activation induces the production and secretion of luteinizing hormone (LH) and follicle-stimulating hormone (FSH), which, in turn, regulate gonadal functions [23,24]. Lately, novel roles for the GnRH peptide in other regions of the CNS have been described, and these are associated with aging, cognition, motor function, and inflammation [25,26,27,28,29]. Particularly in the SN, our group recently reported that GnRH was able to induce functional recovery after neural injury [30,31]. Leuprolide acetate is a synthetic nonapeptide and a gonadotropin-releasing hormone (GnRH) agonist that is less susceptible to enzymatic proteolysis and has a higher affinity for the GnRH receptor, which enhances its biological activity [32]. Because native GnRH has limited clinical applicability due to its rapid degradation, we used leuprolide acetate (LA), a clinically approved GnRH agonist widely used in the treatment of precocious puberty and other reproductive disorders.

There is increasing evidence supporting the neurotrophic properties of LA, making it a promising therapeutic option for conditions such as multiple sclerosis, brain trauma, spinal cord damage, and peripheral nerve injuries [33,34,35,36]. The aim of this study was to analyze the effect of LA treatment on sensory function and DRG in a rat sciatic nerve transection (SNT) model.

## 2. Materials and Methods

### 2.1. Animals

Male Wistar rats weighing between 250 and 300 g were obtained from the vivarium at the Universidad Autónoma de Aguascalientes (UAA). The rats were individually housed under standardized conditions, with food and water available ad libitum. The housing environment was maintained at a constant temperature of 21 °C, a relative humidity of 50%, and a 12:12 h light–dark cycle with artificial illumination. All experiments were conducted during the light phase of the cycle. The rats were randomly assigned to one of three experimental groups: sham-operated control (not damaged and untreated), sciatic nerve transection (SNT) group treated with saline solution, and SNT group treated with LA (SNT + LA), with n = 6–8 animals per group; the sample size was determined using G*power 3.1 software. All experimental protocols and procedures were conducted in strict accordance with the ethical guidelines outlined in the guide for the care and use of experimental animals by the U.S. National Institutes of Health (NIH). All procedures adhered to the animal welfare regulations committee of the UAA (CEADI/UAA/0025/18) and to the ARRIVE guidelines.

### 2.2. Sciatic Nerve Transection and Postoperative Care

The surgical procedure utilized the full-transection model previously described in detail by Hernández-Jasso et al. [35]. Briefly, with the animals under deep anesthesia (ketamine/xylazine 50/10 mg/kg), the sciatic nerve of the left limb was exposed and cut using straight microsurgical scissors. The nerve was then reanastomosed to the distal stump by stitching the epineural layer with microsutures (Atramat, Mexico City, Mexico, PGA surgical sutures, 8-0). The muscle and skin were subsequently sewn. To promote healing and alleviate discomfort, a topical antiseptic was applied, and sodium metamizole (15 mg/kg) was administered intramuscularly (IM) once daily for 3 days. After surgery, the animals were placed in a recovery chamber.

### 2.3. Treatment

Administration of LA (Sigma, St. Louis, MO, USA) was initiated one day after SNT. LA was injected intramuscularly (100 µL) at a dose of 10 µg/kg once daily for three consecutive days; thereafter, IM injections were administered every three days over a five-week period. This treatment protocol was similar to that used in previous experiments conducted in spinal cord and sciatic nerve injury models [33,35]. Animals in the sham and SNT groups received 100 µL of 0.9% sodium chloride solution by intramuscular injection.

### 2.4. Retrograde Axonal Transport Analysis

Five weeks after injury, animals from all experimental groups were anesthetized (ketamine/xylazine 50/10 mg/kg), and their left sciatic nerve was exposed through an incision parallel to the femur and then isolated from the surrounding soft tissue. Later, the peroneal, tibial, and saphenous branches were identified and separated. Using a 5 µL micro-syringe (Hamilton, Biel, Switzerland, 87943) with a 34 G needle (Hamilton, Biel, Switzerland, 207434-10), 2 µL of the anionic tracer (dextran; Invitrogen, Carlsbad, CA, USA, Alexa Fluor 568; 10,000 MW) was injected into each left sciatic nerve. The tip of the needle was initially advanced approximately 1 to 2 mm into each nerve branch, and the volume was infiltrated slowly over approximately 1 min. The needle was left in place for an additional 2 min and then slowly withdrawn to prevent leakage of the injected solution. This procedure was repeated for each branch of the nerve, and then the wound was sutured. The animals were sacrificed 72 h after tracer injection. Each animal was deeply anesthetized with ketamine/xylazine (50/10 mg/kg) and perfused with 150 mL of sterile saline solution (0.9% NaCl). After perfusion, dorsal root ganglia (L4, L5, L6, and S1) were removed and fixed in 4% paraformaldehyde (PFA) (Sigma, St. Louis, MO, USA) and cryopreserved until use. DRGs were sectioned and prepared for immunofluorescence as previously described, and images were captured directly after microscopical analysis [31].

### 2.5. Immunohistochemistry and Fluorescence Microscopy

Three days after tracer injection, the animals were deeply anesthetized with ketamine/xylazine (50/10 mg/kg) and perfused with physiological saline. Tissue, including dorsal root ganglia (DRG) and sciatic nerves, was collected for immunofluorescence analyses. To evaluate gliosis in the injured tissue, which may serve as an indicator of neurological recovery, immunofluorescence analysis of glial fibrillary acidic protein (GFAP) was performed. Additionally, to assess the possible infiltration of macrophages in response to the injury, Iba1 localization was analyzed. Dorsal root ganglia (DRG) and nerve tissues were fixed overnight in 4% paraformaldehyde at 4 °C. The tissues were then transferred to a series of increasing sucrose concentrations (10%, 20%, and 30%) in 0.1 M phosphate buffer (pH 7.4), spending 24 to 36 h at each concentration for cryoprotection prior to sectioning. Following cryoprotection, the sciatic nerves and DRG were embedded in Tissue-Tek O.C.T. compound (Sakura Finetek, Torrance, CA, USA) and mounted onto aluminum sectioning blocks. Fifteen-micrometer-thick sections were obtained using a cryostat (Leica CM3050 S, Buffalo Grove, IL, USA), producing longitudinal sections of the nerves and cross-sections of the DRG. The tissue sections were mounted on silane-treated glass slides to enhance adhesion. The sections were incubated with primary polyclonal antibodies against GFAP and Iba1 (Table 1), diluted in PBS containing 0.05% Triton X-100 and 1% non-fat dry milk, and incubated overnight at 4 °C. The secondary antibodies (Table 1) were diluted 1:1000 in TPBS with 1% non-fat dry milk (Bio-Rad, Hercules, CA, USA), and DAPI (100 ng/mL) (Sigma-Aldrich, St. Louis, MO, USA) was used for nuclear counterstaining. Incubation was carried out for 2 h at room temperature. Control samples without primary antibodies were included in the analysis. Because satellite glial cells closely surround neuronal somata in the DRG, quantitative assessment of GFAP immunoreactivity specifically around neuronal cell bodies can be technically challenging and may lead to apparent neuronal labeling due to the tight anatomical association between these cells. Therefore, to obtain a more reliable assessment of glial reactivity, quantitative analysis of GFAP expression was performed in the nerve root region, where increased GFAP expression in glial cells following nerve injury was consistently reported. Images were captured using an Olympus BX51 fluorescence microscope (Tokyo, Japan) and analyzed with ImageJ 2.16.0 software (NIH, Bethesda, MD, USA).

### 2.6. Morphological Analysis of Iba1-Postive Cells

Iba1 immunostaining was analyzed using the Skeleton Analysis and FracLac plugins in ImageJ (Fiji distribution, NIH, Bethesda, MD, USA). Images were converted to grayscale and then binarized after brightness and contrast adjustment to enhance the visualization of cellular processes. Individual Iba1-positive cells (macrophages) (n = 100) were isolated for subsequent analysis. Binary images were refined using the close and remove outlier functions.

For morphometric evaluation, images were skeletonized and analyzed using the Skeleton Analysis plugin to quantify the number of branches. Fractal analysis was performed using the FracLac plugin with the box-counting method (grid parameter “Num G” = 4), and the parameter obtained was circularity (an indicator of how circular the cell is).

### 2.7. Gene-Expression Quantification by Real-Time Polymerase Chain Reaction (qPCR)

DRG tissue was subjected to total RNA extraction using the TRIzol Reagent (Invitrogen) according to the manufacturer’s protocol. Each sample, consisting of 250 ng of total RNA, was treated with 20 IU of DNase I (Promega, Madison, WI, USA) for 30 min at 37 °C to remove any contaminating genomic DNA. Subsequently, cDNA synthesis was performed using the High Capacity Reverse Transcription Kit with ribonuclease inhibitor (Applied Biosystems, Waltham, MA, USA). Quantitative real-time PCR was conducted on a StepOne Systems machine (Applied Biosystems) using Maxima SYBR Green/ROX qPCR Master Mix (Thermo Scientific, Waltham, MA, USA). Oligonucleotide primers (listed in Table 2) were designed to span exon–exon boundaries whenever possible, utilizing the BLAST primer algorithm (https://www.ncbi.nlm.nih.gov/tools/primer-blast/ accessed on 3 March 2024). Changes in gene expression were assessed using the comparative threshold cycle (CT) method and the 2-ΔΔCT formula [37]. The target genes were normalized to the geometric mean of the mRNA levels of the reference genes Gapdh and Ppia, with the vehicle treatment used as the calibrator.

### 2.8. Hot/Cold Test for Sensory Recovery

Hind paw withdrawal latency to heat stimulation was assessed using the hot plate test [38]. The entire ventral surface of the left hind paw was placed on a hot plate (Panlab Instruments; Harvard Apparatus, Barcelona, Spain) maintained at a fixed temperature of 50 °C, ensuring that the time the rat remained on the plate did not exceed 60 s to prevent damage. Similarly, withdrawal latency to cold stimulation was assessed using a cold plate maintained at a constant temperature of −4 °C. The maximum time the rat was allowed to remain on the cold plate was set to 150 s to avoid freezing damage.

### 2.9. Statistical Analysis

Data are expressed as mean ± SEM. One-way analysis of variance (ANOVA) and Tukey’s and Fisher’s LSD post hoc tests were performed using GraphPad Prism version 9.00 (GraphPad Software, San Diego, CA, USA). Differences were considered significant at *p* < 0.05. All the experimental groups contained 6–8 animals.

## 3. Results

### 3.1. Effect of LA in Sensory Recovery After SNT

**Figure 1:**
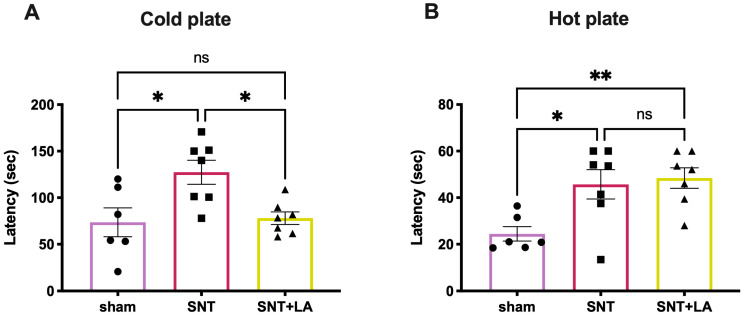
Response to cold and hot in rats with SNT. Latency of response in cold (**A**) and hot (**B**) plate assay in animals with SNT. Results are shown as mean ± SEM from 6 to 8 animals. Experimental groups: sham (control), sciatic nerve transection treated with saline solution (SNT), and sciatic nerve transection treated with leuprolide acetate (SNT + LA). Units are in seconds (s). (*) *p* ≤ 0.05 and (**) *p* ≤ 0.01, as determined by one-way ANOVA with Tukey as post hoc test.

Thermal behavioral tests were used to assess the effect of LA administration, for 5 weeks, on sensory recovery (cold and heat) in SNT-injured rats. Response latency was recorded using the thermal plate test (Figure 1). In the cold sensitivity test (Figure 1A), the sham control group showed a latency of 73.58 ± 15.54 s, which increased significantly to 127.4 ± 12.89 s in the SNT group (*p* ≤ 0.05). In turn, LA treatment induced a significant recovery of the sensory response to cold compared with the lesioned untreated group, as the SNT + LA group showed a reduced latency of 78.01 ± 6.68 s (*p* ≤ 0.05), which was not different from the sham control. In contrast, in the heat sensitivity test (Figure 1B), both injured groups showed a significant increase in latency times (SNT: 45.71 ± 6.30; SNT + LA: 48.44 ± 4.39 s) compared with the sham control group, which presented a latency of 24.45 ± 3.10 s. No differences in heat sensitivity were observed between the LA-treated and the untreated injured groups.

### 3.2. Axonal Transport Recovery After SNT

**Figure 2:**
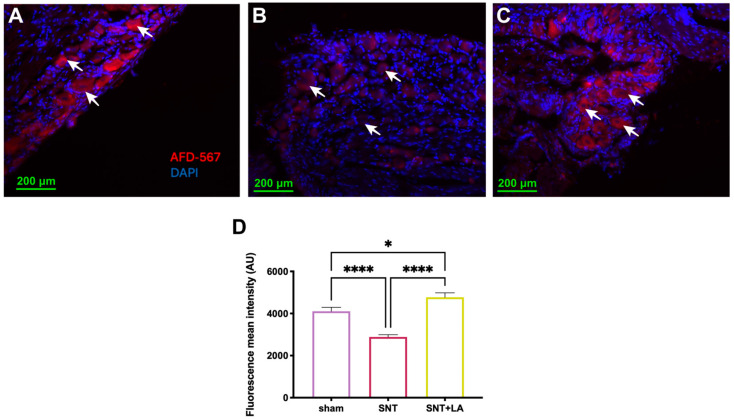
Retrograde axonal transport to DRG. Axonal transport was assessed by Dextran Alexa Fluor-568 injection in the branches of the sciatic nerve 5 weeks after injury. Sections of DRG tissue were analyzed. Representative images at 10× magnification of experimental groups are shown, with a scale bar of 200 μm. Experimental groups: (**A**) sham; (**B**) sciatic nerve transection treated with saline solution (SNT); and (**C**) sciatic nerve transection treated with leuprolide acetate (SNT + LA). (**D**) Analysis of tracer fluorescence in DRG neuronal bodies. Red: Dextran; blue: DAPI staining (nuclei). Arrows indicate positive Dextran neuronal bodies. Results are shown as mean ± SEM from 6 to 8 animals. Units are in arbitrary units (AU). (*) *p* ≤ 0.05 and (****) *p* ≤ 0.0001, as determined by one-way ANOVA with Tukey as post hoc test.

To analyze the functionality of retrograde axonal transport, dextran conjugated to AlexaFluor-568 was injected into the three branches of the sciatic nerve 3 days before tissue collection, and then the presence of red fluorescence in neuronal bodies was analyzed in sections of the DRG (Figure 2A–D). Results indicated that in the sham group, a mean fluorescence intensity of 4105 ± 183.5 arbitrary units (a.u.) was observed (Figure 2A,D). In contrast, the SNT-untreated group exhibited significantly less fluorescence in the somas of sensory neurons of the DRG (2888 ± 104.7 a.u.; *p* ≤ 0.0001) (Figure 2B,D) in comparison to the sham control and LA-treated group. Notably, the SNT + LA group showed a significant recovery of fluorescence in the neuronal bodies, which increased even above the control (4772 ± 208.6 a.u.; *p* ≤ 0.0001) (Figure 2C,D).

### 3.3. Effect of LA in Reactive Gliosis

**Figure 3:**
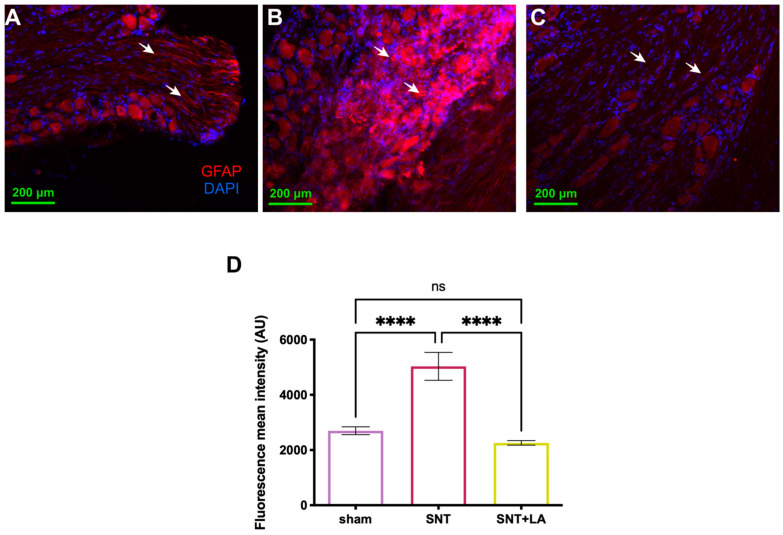
Glial fibrillary acidic protein (GFAP) immunohistochemistry in DRGs. Images at 10× magnification of sections of dorsal root ganglia were analyzed, with a scale bar of 200 μm. Experimental groups: (**A**) sham; (**B**) sciatic nerve transection treated with saline solution (SNT); and (**C**) sciatic nerve transection treated with leuprolide acetate (SNT + LA). (**D**) Analysis of GFAP immunofluorescence mean intensity (red); Blue: DAPI staining (nuclei) in DRG tissue. Arrows show representative immunostaining. Results are shown as mean ± SEM, 6–8 animals. Units are in arbitrary units (AU). (****) *p* ≤ 0.0001, as determined by one-way ANOVA with Tukey as post hoc test.

Figure 3 shows representative tissue sections of dorsal root ganglia (DRG) obtained five weeks after SNT from all experimental groups, which were immunolabeled with a specific antibody directed against GFAP (red) and counterstained with DAPI (blue) (Figure 3A–C). The fluorescence mean intensity of GFAP-IR was significantly increased (*p* ≤ 0.0001) in the SNT group (Figure 3B, 5033 ± 504.5 a.u.) as compared with the sham control (Figure 3A, 2699 ± 143.4 a.u.) and the SNT + LA group (Figure 3C, 2260 ± 81.04 a.u.), which had no differences among them (*p* < 0.05) (Figure 3D).

### 3.4. Iba1-Positive Cells Response to LA in the SNT Model

**Figure 4:**
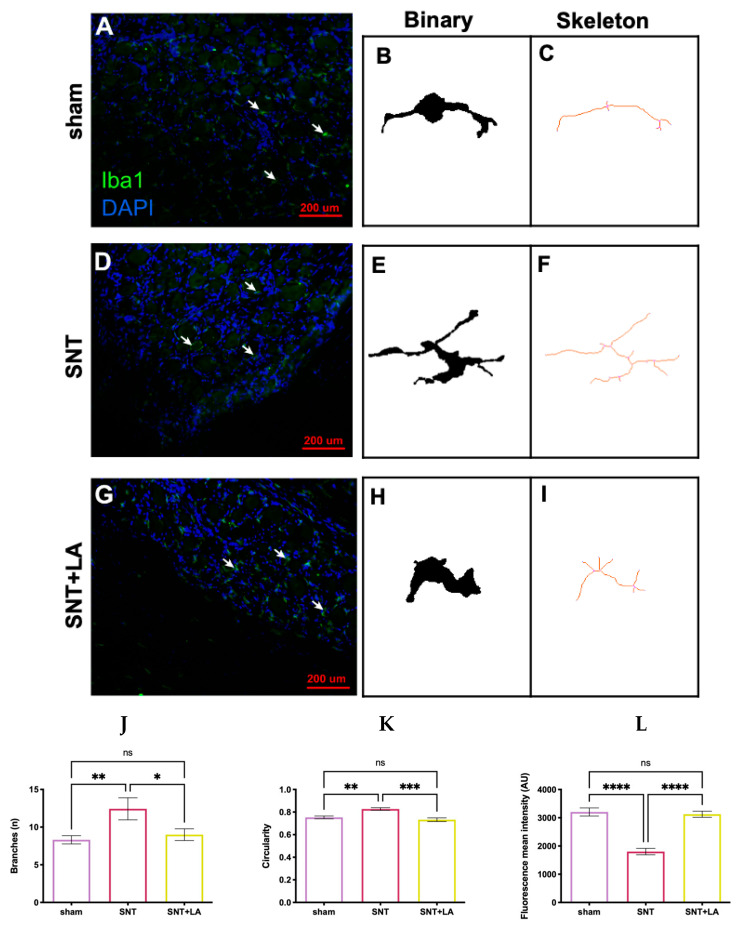
Effect of LA upon SNT-treated DRGs. Ionized calcium-binding adapter molecule 1 (Iba1) immunohistochemistry in dorsal root ganglia tissue was analyzed to assess morphological changes in Iba1-positive immune cells (macrophages). Representative images of dorsal root ganglia of experimental groups, analyzed at 10× magnification, are shown, with a scale bar of 200 μm. Experimental groups: (**A**) sham; (**D**) sciatic nerve transection treated with saline solution (SNT); and (**G**) sciatic nerve transection treated with leuprolide acetate (SNT + LA). (**B**,**E**,**H**) Binary images of the isolated individual Iba-1-postive cells; (**C**,**F**,**I**) skeletonized images of individual cells, where branches are in red. (**J**,**K**,**L**) Analysis of morphological parameters of macrophages and Iba-1 immunofluorescence mean intensity (green); blue: DAPI staining (nuclei) in DRG tissue. Arrows show representative macrophage cells. Results are shown as mean ± SEM, n = 100 Iba1-positive cells, 6–8 animals. Units are in number of branches (**J**), circularity (**K**), and arbitrary units (AU) (**L**). (*) *p* ≤ 0.05, (**) *p* ≤ 0.01, (***) *p* ≤ 0.001, and (****) *p* ≤ 0.0001, as determined by one-way ANOVA with Tukey as post hoc test.

Iba1-positive cell (macrophage) response occurring in the DRG due to the different treatments was studied by immunohistochemistry using a specific antibody directed against Iba1, a commonly used marker to examine macrophage reactivity, by measuring changes in several morphological parameters. Morphological changes were quantified using Iba1 IHC analysis, and macrophage-like IR cells (indicated with arrows) were identified in all treatment groups (Figure 4A,D,G). Individual cells were isolated from the photomicrographs and then converted into binary images (Figure 4B,E,H), allowing for skeleton and fractal analyses. Representative skeleton test images (Figure 4C,F,I) display branches in red. Morphological analysis showed that, in comparison to the sham control (8.3 ± 0.5 branches), the number of branches was significantly increased (*p* ≤ 0.01) in the SNT-untreated group (12.4 ± 1.4 branches), but this effect was clearly reversed (*p* ≤ 0.05) in the SNT + LA group (9.0 ± 0.7 branches) (Figure 4J). A similar result was observed regarding the circularity of the cells (sham: 0.75 ± 0.01; SNT: 0.82 ± 0.01; and SNT + LA: 0.73 ± 0.1 circularity, respectively) (Figure 4K). In contrast, the fluorescence mean intensity of Iba1-IR was significantly reduced (*p* ≤ 0.0001) in the SNT group (1802 ± 112.5 a.u.) as compared with the sham control (3204 ± 144.3 a.u.) and the SNT + LA group (3124 ± 108.3 a.u.), which had no differences among them (*p* < 0.05) (Figure 4L).

### 3.5. Effect of LA on Pro-Regenerative Genes in the SNT Model

**Figure 5:**
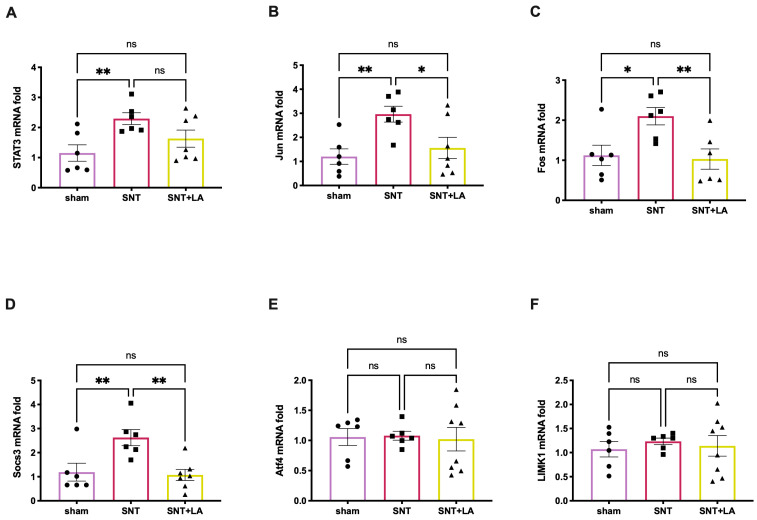
Relative gene-expression of neuroregenerative markers in DRGs. (**A**) Relative change in *Stat3* mRNA; (**B**) relative change in *Jun* mRNA; (**C**) relative change in *Fos* mRNA; (**D**) relative change in *Socs3* mRNA; (**E**) relative change in *Atf4* mRNA, and (**F**) relative change in *Limk1* mRNA. Experimental groups: sham (control), sciatic nerve transection treated with saline solution (SNT), and sciatic nerve transection treated with leuprolide acetate (SNT + LA). Results are shown as mean ± SEM from 6 to 8 animals: (*) *p* ≤ 0.05, and (**) *p* ≤ 0.01, determined by one-way ANOVA with Fisher’s LSD as post hoc test.

To investigate the potential effects of LA upon the activity of pro-regenerative genes, we examined the expression levels of *Stat3, Jun, Fos, Socs3, Atf4,* and *Limk1* in DRG (Figure 5). Following SNT, DRG neurons exhibited a significantly elevated *Stat3* expression (*p* ≤ 0.01) in the injured group (2.294 ± 0.1968 fold) compared to the sham group (1.152 ± 0.2742 fold). Notably, the *Stat3* expression level was slightly lower in the LA-treated group (1.631 ± 0.2851 fold) than in the damaged group (2.294 ± 0.1968 fold) (Figure 5A). A significant induction (*p* ≤ 0.01) of *Jun* expression was also observed in the SNT-untreated group (2.964 ± 0.3296 fold) compared to the sham control group (1.202 ± 0.3190 fold); however, a significant reduction (*p* ≤ 0.05) in *Jun* expression was noted in the LA-treated group (1.561 ± 0.4407 fold) compared to the untreated lesioned group (2.964 ± 0.3296 fold) (Figure 5B). In turn, *Fos*, a gene that is coactivated with *Jun* to facilitate cellular proliferation and differentiation, showed a significantly increased expression (*p* ≤ 0.05) in the SNT-untreated group (2.101 ± 0.2180 fold) compared to the control group (1.123 ± 0.2542 fold). Importantly, the LA-treated group showed a significant reduction (*p* ≤ 0.01) in *Fos* expression (1.030 ± 0.2544 fold), reaching levels comparable to those observed in the sham group (Figure 5C). Similar results were observed for the expression of *Socs3* (Figure 5D). In contrast, the expression levels of *Atf4* and *Limk1* remained unaffected in all three experimental groups (Figure 5E,F).

## 4. Discussion

This study demonstrates for the first time that LA promotes axonal regeneration following SNT. Evidence supporting LA-induced nerve regeneration includes sensory recovery to cold and enhanced axonal tracer transport, as well as a role in modulating the expression of several regeneration- and inflammation-related genes in sensory neurons of DRG.

While the presence of GnRH-R in the central nervous system (CNS) is well-established [39,40,41,42,43,44], its expression in the peripheral nervous system (PNS) has been scarcely reported. It is possible that the observed findings may indicate the participation of GnRH receptors in the DRG, as occurs in the sympathetic ganglia of amphibians, where GnRH modulates sympathetic neurons by inhibiting potassium channels [45,46]; however, this assumption remains to be explored. Previous studies have shown that GnRH analogs, such as LA and buserelin, facilitate functional recovery in rats with SNT, assessed through gait analysis and nerve conduction velocity measurements, effects attributed to GnRH-R activation in motor neurons of the spinal cord [35,47]. GnRH has been associated with the regulation of neurotrophic factor expression, particularly following neural injury. Experimental studies using animal models of spinal cord damage and sciatic nerve transection have reported that sustained GnRH administration enhances the expression of key neurotrophins, including nerve growth factor (NGF), brain-derived neurotrophic factor (BDNF), and neurotrophin-3 (NT-3) [30,31,48]. In addition, these treatments have been linked to improvements in synaptic protein expression and myelin preservation, supporting a potential neurotrophic and synaptogenic role of this hormone. The present study extends these findings by suggesting that peripheral nerve regeneration may occur through the induction of neurotrophic factors such as BDNF and NGF, as previously reported by our research group in spinal cord injury models.

Thermal stimulus encoding differs between cold and heat perception, since heat encoding is gradual; increasing temperatures activate more heat-sensitive neurons and enhance individual neuronal responses [49,50]. In contrast, cold encoding lacks a gradual pattern and may involve combinatorial coding, where specific combinations of activated neurons determine the response to cold stimuli [50]. In our study, LA treatment promoted the recovery of sensory function in response to cold stimulation. This finding suggests that LA may exert neuroprotective or neuroregenerative effects on DRG neurons involved in cold perception, potentially restoring neuronal connectivity and the activation patterns required for proper cold sensation encoding. One possible mechanism underlying this effect is the induction of neurotrophic factors. Previous studies have shown that neurotrophic factors can modulate the expression and functional activity of TRPM8, a cold-sensitive ion channel that plays a key role in sensory neuron signaling [51]. On the other hand, no differences in heat sensitivity were observed here, in contrast to previous studies in which GnRH administration was reported to promote recovery of heat sensitivity [30] in orchiectomized rats. This discrepancy may be explained, first, by the use of intact rats in the present work, since administration of leuprolide acetate (LA) may initially induce an increase in various sex hormones, which could alter the conductivity of thermal signaling related to heat perception [52]. In addition, the greater potency of LA compared with native GnRH [32,53] may lead to a differential activation of receptors involved in the detection of thermal stimuli, whether heat or cold. In this context, further studies are required to elucidate the signaling pathways involved and to clarify the mechanisms underlying the differences observed between heat and cold sensitivity. We could not detect the presence of GnRH receptor in the DRG and sciatic nerve samples, and single-cell RNA-seq studies have reported the absence of its transcript in DRG cells [54]; however, we previously identified its expression in the spinal cord [41]. We are currently investigating the distribution of the receptor that is activated by LA to exert the observed effects.

Proper axonal transport is critical for recovery from peripheral nerve injuries, facilitating the transport of growth factors, essential molecules for neuronal survival, transmission of damage signals to the neuronal soma, and tissue repair [55,56,57,58]. SNT completely abolishes nerve retrograde axonal transport after complete cut of the nerve and triggers regenerative signals associated with Schwann cell de-differentiation [59,60,61]. We assessed the effect of LA on retrograde axonal transport in DRG neurons using a fluorophore-conjugated tracer that was injected directly into the distal portion of the transected sciatic nerve. The untreated SNT group exhibited a significantly reduced presence of fluorescence in sensory DRG neurons, indicating impaired retrograde transport. In contrast, the SNT + LA group showed fluorescence levels comparable to those in the sham group, suggesting that LA fully restored retrograde transport in sensory fibers. This restoration may enhance signaling pathways that promote neuronal survival, regeneration, and functional recovery.

Glial fibrillary acidic protein (GFAP) is a marker for the activation of satellite glial cells and non-myelinating Schwann cells in the DRG [62,63]. Also, GFAP expression is significantly upregulated in response to nerve injury and inflammation [64]. Consistent with these reports, our study showed that SNT induced intense GFAP immunofluorescence in the DRG as compared to the control group. Conversely, LA treatment significantly reduced GFAP immunofluorescence in comparison to the SNT group, indicating that LA attenuated glial activation following nerve injury.

Recent research reveals that macrophages exhibit two phenotypes with distinct functions; the M1 phenotype is associated with proinflammatory responses, whereas the M2 phenotype is linked to anti-inflammatory and neuroprotective functions [65]. Interventions such as exercise [66] and minocycline [67] have been shown to alleviate neuropathic pain by shifting macrophages toward the M2 phenotype in the DRG, promoting anti-inflammatory and neuroprotective effects [68]. Morphometric analysis of Iba1-labeled macrophages in our study revealed that SNT induced morphological changes indicative of activation, including significant increases in branching and circularity, and an important reduction in fluorescence intensity. In contrast, LA treatment restored macrophage morphology to a state analogous to the control group, suggesting that LA may promote a shift toward the M2 phenotype. LA has demonstrated significant therapeutic potential in animal models of neurological damage by promoting neuroprotection and regeneration and modulating the inflammatory response [33,34,69,70,71,72]. Therefore, one possible mechanism of LA action is the induction of macrophage phenotype change to M2, enhancing the release of anti-inflammatory cytokines and neurotrophic factors. Additionally, complete SNT induces a cellular response in DRG neurons, modulating various genes and proteins involved in regeneration and inflammation [73].

*Stat3* is a key factor in neural repair and regeneration, influencing neural stem cell fate, promoting axon regrowth, and modulating the repair function of Schwann cells through transcriptional regulation and mitochondrial function [74,75]. *Stat3* activation has been implicated in neuropathic pain regulation in rodent models [76,77,78], and its inhibition alleviates neuropathic pain following peripheral nerve injury [79]. In our study, and consistent with previous findings, SNT induced a significant elevation of *Stat3* expression in DRG neurons; however, LA treatment slightly reduced *Stat3* expression levels in the lesioned DRG. Although *Stat3* plays complex roles in neuroregeneration, LA does not directly activate the *JAK*/*STAT* pathway; instead, it may modulate this pathway indirectly through neurotrophic factors induced by GnRH-R signaling via G-protein-coupled receptors [80]. These results provide insights into the molecular mechanisms by which LA may modulate *Stat3* expression and contribute to nerve regeneration.

*Socs3* is a negative regulator of the *JAK*/*Stat* signaling pathway and limits neurite growth in sensory neurons. Deletion of *Socs3* enhances early regeneration of DRG axons following sciatic nerve injury [81,82]. Our study found that SNT importantly induced *Socs3* expression in comparison to the controls, while, in contrast, LA treatment led to a significant downregulation of *Socs3* expression, which may facilitate the regenerative capacity of DRG neurons after injury. Upon peripheral nerve injury, a neuroinflammatory response propagates from the DRG to the spinal cord via the *CNTF-Stat3-IL-6* pathway; ciliary neurotrophic factor (CNTF) activates *Stat3*, leading to interleukin-6 (IL-6) production in sensory neurons, contributing to neuropathic pain modulation [83]. LA treatment has been shown to modulate inflammation by suppressing cytokine expression [69]. Previous studies have shown that STAT3 signaling may persist for extended periods after injury [84,85]. These findings suggest that STAT3 activation is not exclusively restricted to the initial phase of regeneration, but may also contribute to longer-term regenerative and plasticity-related processes. In this context, the expression changes observed several weeks after sciatic nerve transection in the present study may reflect molecular mechanisms involved in the maintenance and consolidation of the regenerative response rather than only the early regenerative phase. Notably, our findings suggest that LA treatment may facilitate the resolution of the injury by promoting a regenerative phenotype in which, at this later time point, STAT3 signaling is no longer required. In contrast, in untreated rats, the sustained elevation of *Stat3* expression may reflect a prolonged inflammatory state that could even contribute to the establishment of a chronic pain-associated phenotype [86,87]. Therefore, modulating the JAK/STAT pathway, either by downregulating *Socs3* to enhance neuronal regeneration or influencing *CNTF-Stat3-IL-6* signaling to manage neuropathic pain, represents a potential therapeutic strategy following peripheral nerve injury.

Elevated levels of *Jun* mRNA and protein expression in DRG neurons have been reported following axotomy, reflecting activation of injury-related genes [88,89]. In our study, *Jun* expression was upregulated in the SNT group, whereas LA treatment led to a significant decrease in *Jun* expression. *Jun* expression in axotomized neurons is transient and decreases as regenerating nerve fibers reach their peripheral targets [90,91]. The reduction in *Jun* expression observed with LA treatment may be attributed to the restoration of sensory fibers facilitated by LA, indicating progression in nerve regeneration.

*Fos*, a transcription factor involved in DRG regeneration [92,93], is associated with pathological damage and disrupted neuronal functions following peripheral nerve injury. We observed a significantly increased *Fos* expression in the injured group, consistent with previous reports. Treatments such as calcium channel blockers suppress *Fos* expression [94], and inhibition of *Fos* is linked to reduced neuronal damage and improved function [95]. Our findings showed a significant reduction in *Fos* expression in DRG neurons following LA treatment, suggesting a potential neuroprotective effect of LA.

*Atf4* regulates the expression of genes involved in stress adaptation, metabolism, and apoptosis, playing critical roles in tissue development and synaptic plasticity. Recent studies have implicated *Atf4* in heat nociception through non-transcriptional mechanisms [96]. In our study, *Atf4* expression remained unchanged following SNT and LA treatment, correlating with the observed lack of recovery in heat avoidance. Similarly, *Limk1*, known to regulate axonal regeneration after injury [97], showed no significant changes in gene expression, suggesting that LA’s regenerative effects may not involve changes in *Atf4* or *Link1* activation.

## 5. Conclusions

In summary, LA treatment following SNT in male rats promotes recovery of cold sensitivity, enhances retrograde axonal transport, modulates the inflammatory response, and affects the expression of pro-regenerative genes (*Stat3*, *Jun*, *Socs3*, and *Fos*), suggesting its potential contribution to sensory function restoration. The lack of effect on heat sensitivity and unchanged expression of *Atf4* and *Limk1* indicate that LA’s regenerative effects may be specific to certain neuronal populations and pathways. Further research is necessary to elucidate the molecular and cellular mechanisms by which LA facilitates nerve regeneration and functional recovery.

## Figures and Tables

**Table 1:** Antibodies.

Target	Host/Type	Dilution	Source	Cat. No.
GFAP	Rabbit/polyclonal	1:500	Abcam (Cambridge, UK)	Ab16997-1
Iba1	Goat/polyclonal	1:500	Abcam (Cambridge, UK)	Ab5076
Rabbit IgG	Goat/Alexa fluor 594	1:1000	Invitrogen (Carlsbad, CA, USA)	A11012
Goat IgG	Rabbit/Alexa fluor 488	1:1000	Invitrogen (Carlsbad, CA, USA)	A-11078

**Table 2:** Oligonucleotides used reverse transcriptase quantitative PCR (RT-qPCR).

Target	Sequences (5′–3′)	
	Forward	Reverse
*Stat3*	TGGATGCGACCAACATCCTG	CAATGGTATTGCTGCAGGTCG
*Jun*	GCACATCACCACTACACCGA	GGGAAGCGTGTTCTGGCTAT
*Socs3*	CTACGCATCCAGTGTGAGGG	TGAGTACACAGTCGAAGCGG
*Atf4*	GGTGGCCAAGCACTTGAAAC	GTCCCGGAAAAGGCATCCTC
*Fos*	TACTACCATTCCCCAGCCGA	GCTGTCACCGTGGGGATAAA
*Limk1*	ATCACAGAGTACACTAAGGGC	GTTCATGCAATGGAGGTAGGC
*Gapdh*	TGTGTCCGTCGTGGATCTGA	CTTCACCACCTTCTTGATGTCACT
*Ppia*	GGTTCCTCCTTTCACAGAAT	AATTTCTCTCCGTAGATGGAC

## Data Availability

The data presented in this study are available on request from the corresponding authors due to departmental policy.

## Abbreviations

The following abbreviations are used in this manuscript:

a.u.	Arbitrary units
BDNF	Brain-derived neurotrophic factor
CNS	Central nervous system
CNTF	Ciliary neurotrophic factor
DRG	Dorsal root ganglia
FSH	Follicle-stimulating hormone
GnRH	Gonadotropin-releasing hormone
LA	Leuprolide acetate
LH	Luteinizing hormone
NGF	Nerve growth factor
SN	Sciatic nerve
SNT	Sciatic nerve transection
